# Psychological implications of unemployment among higher educated migrant youth in Kolkata City, India

**DOI:** 10.1038/s41598-024-60958-y

**Published:** 2024-05-03

**Authors:** Mohai Menul Biswas, Kailas Chandra Das, Illias Sheikh

**Affiliations:** 1 Department of Migration and Urban Studies, International Institute for Population Sciences, Mumbai, 400088 India; 2 Department of Extra Mural Studies, International Institute for Population Sciences, Mumbai, 400088 India

**Keywords:** Psychology, Health care

## Abstract

Mental health issues are intricately linked to socioeconomic background, employment and migration status. However, there remains a gap in understanding the mental health challenges faced by graduate youth in India, particularly in Kolkata City. This study aims to assess the prevalence and associated risk factors of depression, anxiety, and stress among higher-educated migrant youth. A survey was conducted on four hundred migrant graduate youths aged 21–35 residing in Kolkata. Measures included socio-demographics and the Depression Anxiety Stress Scale (DASS-21). Chi-square tests and binary logistic regression were employed to identify factors associated with mental health issues. The overall prevalence rates were 54.4% for depression, 61.8% for anxiety, and 47.9% for stress. Unemployed youths exhibited significantly more symptoms of depression and anxiety than their employed counter parts. The logistic regression model showed that unemployed youth, female sex, never married, and second- and third-time migrant youths were risk factors for high scores on the DASS-21. This study showed that mental health issues were alarming in the higher educated migrant youth. The study suggests the implementation of skill-based, job-oriented, and professional courses at the graduation level to prevent graduates from being rendered unproductive and jobless. Beside these, regular psychological support should be provided to the higher educated youth by the local governments.

## Introduction

The psychological problems induced by unemployment are widely recognized, with research indicating that increasing unemployment rates correlate with a higher incidence of health issues, particularly those related to mental health^1^. This issue is a source of concern for both developed and developing countries. India, as a developing nation, grapples with the persistent challenges of unemployment and underemployment, despite ongoing policy emphasis and programs aimed at addressing and eliminating these issues^2^. Despite various initiatives, the unemployment rate is not only on the rise in the country but also on an all-time high. The Periodic Labour Force Survey (PLFS), conducted by the National Sample Survey Office (NSSO), revealed a concerning trend. Despite an increase in education levels, there was a decline in non-agricultural job growth. Between 2011–2012 and 2017–2018, employment in India dropped from 474 to 465 million. Simultaneously, the unemployment rate witnessed a significant surge, reaching an all-time high of 6.1% in 2017–2018, compared to the 3% recorded in 2011–2012^3^. The most alarming concern emerged from the substantial increase in joblessness among educated youth, nearly doubling from 6.1% in 2011–2012 to a staggering 17.8% in 2017–2018 across all categories. Specifically, the unemployment rate among higher-educated youth experienced a significant rise, surging from 19.2 to 35.8%, while postgraduates faced an increase from 21.3 to 36.2%. Equally disheartening is the figure for unemployment rates among the youth aged 15–29 years, a demographic that constitutes approximately 27–28% of the population (PLFS, Ministry of Statistics and Programme Implementation (MoSPI), 2019)^3^. The pervasive issue of unemployment among highly educated youth, particularly in the Indian context, remains a major concern and subject of extensive discussion; however, it has yet to witness significant resolution.

Recent studies have shown that situational factors leading to either unemployment or underemployment appear to be associated with the risk of common mental health consequences, such as depression, anxiety, and stress^4,5^. Underemployed or unemployed individuals often feel neglected and frustrated, which may lead to psychiatric suffering. In extreme cases, they can develop drug addictions and engage in criminal activities^6^. Many previous studies have reported that mental suffering, including depression, anxiety disorders, stress, hopelessness, and panic attacks, is associated with underemployment and unemployment. These mental health issues arise due to factors such as increased competition, joblessness, job insecurity, low wages, and a lack of opportunities to practice acquired skills^4–10^.

Some researchers have formulated that higher education leads to higher unemployment, resulting in substantial mental health issues. The research paper, published in The Lancet Public Health, is the first to provide evidence of higher levels of depression and anxiety among higher education youths compared to their peers. The authors found that by age 25, these difference had disappeared between graduates and non-graduates. This phenomenon may be related to increased financial pressures and concerns about achieving high results within the broader economic and social context^11^. Moreover, family and social pressures associated with job-seeking activities, along with higher expectations from university graduates, also act as potential mediators of depression and stress disorders among university graduates exploring the job markets^12,13^. Unemployed youth report higher mental health problems compared to employed youth^14,15^. A study found that unemployed persons and those actively seeking jobs have a higher risk of depressive symptoms than individuals who have found employment. In parallel, socio-demographic characteristics, such as being female, unmarried, experiencing a lower quality of life, having a lower family socioeconomic status, academic major, and willingness to accept irregular employment, are also significantly associated with the risk of mental disturbances^5,12,16^. On the other hand, research also highlights that mental health problems can lead to unemployment. Considerable research indicates a strong association between unemployment and common mental health issues such as depression and anxiety^17,18^. Evidence suggests that unemployment both leads to increased anxiety and depression and that heightened anxiety and depression can, in turn, predict future unemployment^18,19^.

Jahoda^20^ posited that employment shapes daily routines, serving as a crucial latent function. Unemployment disrupts established time structures, potentially harming psychological well-being. Employment fosters shared experiences, widening social connections beyond the family. Structured activity from employment is vital for psychological well-being, further highlighting the challenges faced by the unemployed^20^. Unemployed individuals engage in fewer activities, lacking support, risking lower well-being. Employment provides societal position, status, and identity, crucial for mental health. The absence of these elements in unemployment can erode both status and identity, leading to diminished well-being.

The impact of human migration on mental health is complex and varies across cultures^21^. Migrant employees report a higher risk of developing adverse mental health conditions than non-migrant employees^21–23^. While there are common mental health problems for both migrant and non-migrant employees, differences in the nature and intensity of their experiences are apparent. Migrants often face stress related to financial, familial, living, and other factors, particularly among youth^23,24^. Li Chen et al.'s study found that up to 50% of unemployed migrant workers are mentally unhealthy. Another study revealed that migrant employees (15.3%) had a slightly higher prevalence of depression than non-migrant employees (12.0%)^25^. Notably, studies focusing on higher-educated migrant youth experiencing unemployment-induced mental health issues are scarce in existing research.

Studies indicate a higher risk of serious mental health problems in urban metro areas compared to rural regions^26,27^. This present study, conducted in Kolkata, the educational and coaching hub of West Bengal, explores the mental health dynamics in a densely populated metropolitan city with significant immigration. Kolkata, the capital of West Bengal, experiences a substantial influx of youth seeking educational opportunities but faces limited job prospects due to modest industrial development and slow private sector growth^28^. The Economic Survey of 2006–2007 notes a decline in organized sector employment growth, contributing to a high unemployment rate in Kolkata^29^. This phenomenon is attributed to labor force migration to the city in search of job opportunities, a common trend in developing economies. Hence, the study focuses on mental health in the capital city of West Bengal, Kolkata.

In West Bengal, the situation mirrors global and national trends. A PLFS report reveals that approximately 25.7% of youths (15–29 years) with tertiary education were unemployed in the 2017–2018 economic year, aligning with national figures^3^. Despite being qualified for permanent jobs, millions of university graduates struggle to secure employment, as the job market fails to expand rapidly enough to accommodate the growing number of graduates^30^. The socio-cultural structure in West Bengal steers university graduates towards government employment rather than entrepreneurship. However, limited vacancies and a large pool of aspirants result in the majority failing to secure coveted positions, impacting productivity and potentially leading to reduced self-esteem or serious mental health issues. Additionally, many employed higher-educated youth experience underemployment and contractual positions, leading to mental health challenges due to heavy workloads and job insecurity in both public and private sectors.

A recent systematic review focused on West Bengal revealed a 11.8% prevalence of current mental morbidity and 15.1% for lifetime mental morbidity, including any mental disorder, alcohol abuse, and dependence^31^. News reports highlight suicides linked to unemployment among educated individuals in and outside West Bengal^32^. With no studies on mental health among higher-educated migrant youth, the chosen study area and objectives are justified. The current study adopts the null hypothesis of having no relationship between employment status and mental health problems. It aims to assess mental health status among higher-educated migrant youth and identify associated risk factors.

## Data and methods

### Study design and data collection

A cross-sectional study was conducted among graduate youths aged 21–35 years residing in the city of Kolkata. Data were collected during July and October 2022 through random sampling method. A total of 400 respondents participated in this study, and out of those 395 were kept for final analysis, considering the incomplete responses.

### Participants and procedure

First, the investigator met the sampled participants personally and explained the purpose of the present study. In some situations, permission was also taken from the concerned authorities such as schools and other educational institutions. Then after, the investigator established rapport with the participants and requested them to participate voluntarily and cooperate in the data collection process and assured them that their responses would be kept confidential and utilized for research purposes only. The participants were asked to read the instructions carefully given on the top. They were also requested to answer all the statements given in the scales sincerely. After taking the consent, data was obtained separately from each of the participants. The background and purpose of the research endeavor was explained thoroughly to each participant so that every answer is truthful, reliable and transparent.

### Description of variables

#### Independent

##### Socio-demographics

Questions pertaining to socio-demographics included sex (male or female), age, permanent residence (urban or rural), educational status (graduation, post-graduation and M.Phil./PhD), employment status (employed or unemployed), relationship status (i.e., single, engaged in relationship, or married), religion (Hindu/Muslim/Christians), and socioeconomic status. Socioeconomic status was measured using total monthly family income, which was categorized into higher, middle, and lower income groups corresponding to monthly family income of > INR 50,000, INR 50,000 to INR 25,000, and < INR25000, respectively.

#### Migration

For measuring the use of migration related phenomena, questionnaire included the following questions—place of permanent residence (urban or rural) and time since migration (months to year). Further questions asked about ‘migration accompany person (alone/with family/with friend/relative/others).

### Dependent

#### Depression anxiety stress scale (DASS)

Depression, anxiety, and stress were assessed utilizing the Depression Anxiety Stress Scale (DASS-21)^29^ comprising 21 items and three dimensions (seven items per dimension). These three dimensions cover (1) “I could not seem to experience any positive feeling at all” for depression; (2) “I was worried about situations in which I might panic” for anxiety; and (3) “I found it difficult to relax” for stress. The responses were recorded on a four-point Likert scale ranging from 0 (Did not apply to me at all) to 3 (Applied to me very much, or most of the time—Almost always). Higher scores on each dimension reflect higher depression, anxiety and stress respectively. The sub-scales are scored as follows: For depression, the categories are normal (0–9), mild (10–13), moderate (14–20), severe (21–27), and extremely severe (+ 28); for anxiety, the categories are normal (0–7), mild (8–9), moderate (10–14), severe (15–19), and extremely severe (+ 20); and for stress, the categories are normal (0–14), mild (15–18), moderate (19–25), severe (26–33), and extremely severe (+ 34) (29).

Cronbach’s alpha, α or coefficient alpha was used to measure the internal consistency among the outcome variables, depression, anxiety and stress (DASS). The Cronbach’s alpha measure shows the very high level of internal consistency among the various items of DASS. The overall Cronbach’s alpha of 0.83 indicates good or acceptable reliability.

#### Data compilation and analysis

The data analyses were performed using the statistical software STATA version 16.0. In the present study, moderate, severe, and very severe were combined to calculate scores of depression, anxiety, and stress on the DASS^3^. Descriptive statistics (e.g., frequencies, percentages) were performed to present the socio-demographic, migration characteristics and mental health problems. The cross tabulations with Chi-square test (χ^2^) was used to show the association between various attributes. All significant variables in the bivariate tests were entered into a binary logistic regression with "depression”, “anxiety” and “stress” as the dependent variables. The results of the binary logistic regression were interpreted with 95% confidence intervals, and a p-value less than or equal 0.05 was considered significant.

### Ethics

This study was approved by the Students Research Ethics Committee (SREC) of the International Institute for Population Sciences (IIPS), Mumbai, India. [ref. no: IIPS/ACAD/MMB/IO-210/2022]. Verbal and formal written informed consent from all respondents were obtained before participating in the study. All subjects were also informed about the (1) nature and purpose of the study, (2) procedure of the study, (3) right to refuse, and (4) right to withdraw from participating in the study. The study did not include any minor participants. The study also did not involve human participants including minors (including the use of tissue samples). The participants did not gain any financial benefit from taking part in the study. Confidentiality of data and anonymity to the participants were ensured. In addition to this, all methods were performed in accordance with the relevant guidelines and regulations of SREC, IIPS.

### Operational definition

#### Higher education

According to All India Survey on Higher Education “Higher education is tertiary education leading to award of an academic degree which is obtained after completing 12 years of schooling”^28^. In this study, we have adopted at least an undergraduate degree holder in any discipline as higher educated.

#### Youth

Youths were defined as individuals aged 13–35 years according to the National Youth Policy^33^. In the present study, we have considered individuals aged 21–35 years as youth because most of the students graduate at the age of 21 years and in most cases cross the age of 30 by the time they get a job.

#### Employed

Persons who are engaged in any economic activity. Here we have considered higher educated employed migrant youths (21–35 years), which means an individual holding at least an under graduate degree who is currently working and has migrated from their usual place of residence.

#### Unemployed

Unemployed persons are those who are seeking or available for work. In this study, we have considered higher educated unemployed migrant youth, which means those individuals who are at least an undergraduate degree holder and looking for job.

#### Migrants

Migrants comprise individuals who changed their place of usual residence.

#### Duration of migration

The 64th round of National Sample Survey (NSS) defined short term migrants as who had stayed away from their place of origin for a period of 1 month or more but less than 6 months during the last 365 days and long-term migration for a duration of more than six months or more^34^.

#### Incidence of migration

Incidence of migration means number of times they migrated during their education or job.

## Results

### Study participants

Among the overall 395 study participants, almost two-third (254) were unemployed (Table 1). Majority of the study participants were male (58.7%), from rural areas (80%), belonged to the age group of 26–30 years (46%) and were unmarried (49.4%). Almost half of the youths hold graduation degree followed by postgraduation (44.3%), whereas 5.57% (22) had MPhil/PHD degree. As far as socio-religious groups were concerned, Hindu represented almost 60% of the study population, 37.9% were Muslims, and Other Backward Group (OBC) comprised of 41.7% of the study population.

**Table 1:** Distribution of variables among respondents by depression, anxiety and stress.

Variables	Depression		Anxiety		Stress		Total (N = 395)
	Yes (%)	*P*-value	Yes (%)	*P*-value	Yes (%)	*P*-value	
Sex							
Male	123(53.02)	0.501	128(55.17)	0.001	106(45.69)	0.306	232(58.73)
Female	92(56.44)		116(71.17)		83(50.92)		163(41.27)
Type of sample							
Unemployed	168(66.14)	0.000	177(69.69)	0	115(45.28)	0.170	254(64.3)
Employed	47(33.33)		67(47.52)		74(52.48)		141(35.7)
Permanent residents							
Rural	175(55.38)	0.449	196(62.03)	0.836	150(47.47)	0.763	316(80)
Urban	40(50.63)		48(60.76)		39(49.37)		79(20)
Age group							
21–25	84(59.15)	0.000	89(62.89)	0.014	50(35.21)	0.001	142(35.95)
26–30	107(59.12)		121(66.85)		102(56.35)		181(45.82)
31–35	24(33.33)		34(47.22)		37(51.39)		72(18.23)
Educational qualification							
Graduation	94(47.47)	0.020	111(56.06)	0.050	84(42.42)	0.082	198(50.13)
Post-graduation	108(61.71)		119(68.00)		92(52.57)		175(44.30)
M.PHIL/Ph.D	13(59.09)		14(63.64)		13(59.09)		22(5.57)
Marital status							
Never married	128(65.98)	0.000	133(68.56)	0.049	83(42.78)	0.244	194(49.11)
Married	45(34.09)		71(53.79)		70(53.03)		132(33.42)
In a relationship/engaged	42(60.87)		40(57.97)		36(52.17)		69(17.47)
Religion							
Hindu	128(53.56)	0.874	150(62.76)	0.436	130(54.39)	0.003	239(60.51)
Muslim	84(56.00)		89(59.33)		58(38.67)		150(37.97)
Christians	3(50.00)		5(83.33)		1(16.67)		6(1.52)
Monthly family income							
Lower income	78(58.21)	0.054	94(70.15)	0.027	60(44.78)	0.677	134(33.92)
Middle income	95(57.58)		99(60.00)		82(49.70)		165(41.77)
Upper income	42(43.75)		51(53.13)		47(48.96)		96(24.30)
Migration related variable							
Duration of migration							
Short term migration	16(57.14)	0.765	19(67.86)	0.492	9(32.14)	0.084	28(7.09)
Long term migration	199(54.22)		225(61.31)		180(49.05)		367(92.91)
Incidence of migration							
First time	56(55.45)	0.548	58(57.43)	0.110	39(38.61)	0.049	101(25.57)
Second time	105(56.45)		125(67.20)		90(48.39)		186(47.09)
Third time and above	54(50.00)		61(56.48)		60(55.56)		108(27.34)
Migration accompany							
Alone	131(62.09)	0.001	133(63.03)	0.581	98(46.45)	0.55	211(53.42)
Family/Friends	84(45.65)		111(60.33)		91(49.46)		184(46.58)
Total(N)	215(54.43%)		244(61.77%)		189(47.85%)		395(100)

### Prevalence of depression, anxiety and stress

**Figure 1:**
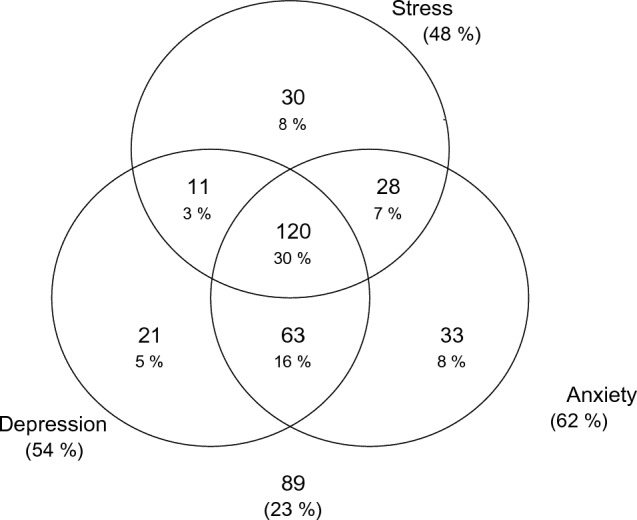
Venn diagram showing mental health issues in the study sample.

The overall prevalence of depression, anxiety and stress among the study participants were 54.4% (n = 215), 61.8% (n = 244) and 47.8% (n = 189) respectively. Thirty percent of participants experienced all the three symptoms mental health (Fig. 1).

### Socio-demographic characteristics and mental health issues

The prevalence of depression (56.44%), anxiety (71.17%) and stress (50.92%) was comparatively higher among female than their counterparts (Table 1). Sex was statistically associated with the anxiety (χ^2^ = 10.371, *p* = 0.001) and it was not significant for depression (χ^2^ = 0.453, *p* = 0.501) and stress (χ2 = 1.05, *p* = 0.306). Unemployed youths reported significantly higher symptoms of depression (66.1% vs. 33.3%; χ^2^ = 39.3, *p* = 0.000) and anxiety (69.7% vs. 47.5%; χ^2^ = 18.9, *p* = 0.000) compared to employed youths, whereas the symptoms of stress were more among employed youths than their counter parts (45.3% vs. 52.5%; χ^2^ = 1.89, *p* = 0.17). Age group was significantly associated with the symptoms of depression, anxiety and stress, and the prevalence was higher among the age group of 26–30 years except stress. Educational status of the respondents was also significantly associated with depression (χ^2^ = 7.798, *p* = 0.02) and anxiety (χ^2^ = 5.642, *p* = 0.050), and it was not significant for stress (χ^2^ = 5.013, *p* = 0.082). Moreover, the prevalence of depression, anxiety and stress was comparatively higher among the MPhil/PhD holders and the post-graduates than graduate youths. Never-married youths experienced higher level of depression (66%) and anxiety (68.6%) compared to other groups, whereas the prevalence of stress was comparatively higher among married youths (53%). Moreover, the symptoms of stress were significantly associated with religion. The study also found that there was significant association between monthly family income status and symptoms of anxiety (χ^2^ = 7.241, *p* = 0.027). The individuals coming from lower- and middle-income families reported higher symptoms of depression and anxiety whereas there was no significant association between stress and monthly family income (χ^2^ = 0.78, *p* = 0.677).

### Migration characteristics and mental health issues

The study found that most of the participants were long term migrants (92.9%) (Table 1). The prevalence of depression (57.1% vs. 54.2%) and anxiety (67.9% vs. 61.3%) was slightly higher among short-term migrants compared to their counterparts, whereas long-term migrants were experiencing higher burden of stress (49.1% vs. 32.1%). Moreover, the prevalence of depression (45.6%) and anxiety (60.3%) was comparatively lower among the youths who migrated with their family members.

### Risk factors mental health problems

Unemployed migrant youths were more likely to have depression [AOR with 95% CI 3.72 (1.94–7.16)] and anxiety [AOR with 95% CI 3.01 (1.55–5.86)] compared to employed youths (Table 2). Females have significantly higher odds of experiencing anxiety [AOR with 95% CI 2.95 (1.76–4.95)] compared to males. As far as educational status was concerned, the Post-graduate youths were experiencing a higher burden of depression [AOR with 95% CI 2.1 (1.26–3.49)] compared to graduate migrant youths. The age group "26–30" had higher odds of experiencing stress [AOR with 95% CI 2.05 (1.13–3.75)] compared to the age group "21–25". Never-married/Single migrant youths had three times and two times higher prevalence of depression and anxiety, respectively, compared to married youths. However, the duration of migration status did not show any significant association with mental health, but the incidence of migration played an explanatory role in the occurrence of depression and anxiety. Second-time and third-time migrant youths had higher odds of experiencing depression [AOR with 95% CI 1.58 (1.13–3.03)] and [AOR with 95% CI 2.31 (1.15–4.47)] respectively. It was also found that second-time and third-time migrant youths had higher burden of anxiety [AOR with 95% CI 2.79 (1.11–47.01)] and [AOR with 95% CI 1.91 (0.66–5.53)] respectively, compared to first-time migrants.

**Table 2:** Binary regression analysis of the variables by depression, anxiety and stress.

Background characteristics	Depression		Anxiety		Stress	
	Unadjusted odds ratio	Adjusted odds ratio	Unadjusted odds ratio	Adjusted odds ratio	Unadjusted odds ratio	Adjusted odds ratio
Employment status						
Employed®						
Unemployed	3.91***(2.53 6.04)	3.72***(1.94 7.16)	2.54***(1.66 3.88)	3.01***(1.55 5.86)	0.75 (0.5 1.13)	1.04 (0.56 1.95)
Place of permanent resident						
Urban area®						
Rural area	1.21 (0.73 1.98)	1.15 (0.63 2.09)	1.05 (0.63 1.74)	1.07 (0.59 1.93)	0.92 (0.56 1.51)	1.18 (0.68 2.07)
Sex						
Male®						
Female	1.15 (0.77 1.72)	1.56*(0.94 2.6)	2.01***(1.31 3.07)	2.95***(1.76 4.95)	1.23 (0.83 1.84)	1.33 (0.83 2.14)
Educational status						
Graduation®						
Post Graduate	1.78***(1.18 2.7)	2.1***(1.26 3.49)	1.67**(1.09 2.54)	1.52 (0.92 2.5)	1.5*(1 2.26)	1.18 (0.74 1.89)
M.Phil./Ph.D	1.6 (0.65 3.91)	2.02 (0.65 6.28)	1.37 (0.55 3.42)	1.16 (0.38 3.57)	1.96 (0.8 4.8)	1.85 (0.67 5.11)
Age Group						
21–25®						
26–30	1 (0.64 1.56)	1.13 (0.6 2.11)	1.2 (0.76 1.9)	1.56 (0.81 3.01)	2.38***(1.51 3.74)	2.05**(1.13 3.75)
31 & above	0.35***(0.19 0.62)	1.4 (0.54 3.63)	0.53**(0.3 0.95)	1.3 (0.5 3.36)	1.95**(1.09 3.46)	1.58 (0.64 3.88)
Marital status						
Married®						
Never married/Single	3.75***(2.35 5.98)	3.02**(1.04 8.77)	1.87***(1.19 2.96)	0.85 (0.29 2.53)	0.66*(0.42 1.03)	1 (0.37 2.69)
In a relationship/Engaged	3.01***(1.65 5.49)	2.09 (0.73 6)	1.19 (0.66 2.13)	0.58 (0.2 1.72)	0.97 (0.54 1.73)	1.43 (0.53 3.84)
Religion						
Hindu®						
Muslims	1.10 (0.73 1.66)	1.14 (0.64 2.03)	0.86(0.56 1.31)	0.93 (0.52 1.65)	0.52 (0.34 .80)	0.78 (0.46 1.33)
Christians	0.86 (0.17 4.38)	1.09 (0.17 6.91)	2.96 (0.34 8.65)	5.33 (0.53 53.89)	0.16 (0.01 1.45)	0.21 (0.02 2.02)
Family income						
Upper income®						
Lower income	1.79**(1.05 3.04)	0.91 (0.47 1.76)	2.07***(1.2 3.58)	1.73 (0.89 3.36)	0.85 (0.5 1.43)	1.06 (0.57 1.99)
Middle income	1.74**(1.05 2.9)	1.55 (0.86 2.8)	1.32 (0.8 2.2)	1.35 (0.76 2.41)	1.03 (0.62 1.7)	1.07 (0.61 1.86)
Duration of migration						
Short term migration®						
Long term migration	0.89 (0.41 1.93)	1.32 (0.53 3.3)	0.75 (0.33 1.7)	1 (0.37 2.72)	2.03(0.9 4.61)	1.64 (0.65 4.14)
Incidence of migration						
First time®						
Second time	1.04 (0.64 1.7)	1.58**(1.13 3.03)	1.52 (0.92 2.5)	2.79**(1.11 7.01)	1.49 (0.91 2.44)	1.1 (0.61 1.98)
Third time	0.8 (0.47 1.38)	2.31**(1.15 4.47)	0.96 (0.56 1.66)	1.91* (0.66 5.53)	1.99**(1.14 3.45)	1.68 (0.81 3.48)
Migration accompany						
With Family/Friends®						
Alone	1.95***(1.3 2.91)	1.01 (0.49 2.08)	1.12 (0.75 1.68)	1.11 (0.54 2.31)	0.89 (0.6 1.32)	0.82 (0.41 1.66)

(R) = Reference Category. *p < 0.05, **p < 0.01, ***p < 0.001.

## Discussion

Mental health issues are a pervasive global concern, particularly among higher-educated migrant youth, whose well-being can be significantly influenced by their employment status. The intersection of educational attainment, migration status, and employment status presents unique stressors and challenges that can adversely affect their mental health. Unemployment among educated youth stands out as a significant social problem in India, especially in cities like Kolkata, where the rates of unemployment have continued to rise. This trend is likely attributed to inadequate job creation despite sustained economic growth over the past several years^35^. Remarkably, the exploration of mental health issues among higher-educated migrant youth in Kolkata City has been notably lacking in previous studies. The present research aims to bridge this gap by investigating the potential connections between psychological distress and the employment status of higher-educated migrant youths.

Nearly half of the population has reported experiencing mental health issues such as depression, anxiety, and stress, with higher rates of these conditions observed among higher-educated youths^4,5^. The findings underscore that unemployed individuals face significantly higher odds of encountering depression and anxiety compared to their employed counterparts. As no other studies specifically focus on higher educated migrant youth in India, particularly in the capital city of West Bengal, Kolkata, making rigorous comparisons to depict mental health status and its contributing factors becomes challenging. The sole study conducted among unemployed and employed youth in the Kashmir Valley revealed twice as high scores for all three mental health indicators compared to the employed groups^13^. A study in Bangladesh also supports our findings, demonstrating elevated mental health issues among unemployed youths^4^.

Various previous studies have outlined reasons behind heightened mental health issues among the unemployed. Factors such as joblessness, economic instability, lack of social identity, financial dependency, feelings of worthlessness, and low self-esteem have emerged as key contributors to mental health challenges among the unemployed^7–12^. In our study location, limited job opportunities in both government and non-government sectors stand out as one of the leading causes of unemployment, consequently contributing to mental health issues^28^. Another study conducted in West Bengal emphasized the absence of industrial setups, leaving higher-educated groups vulnerable by not providing sufficient job opportunities^35^. Notably, the prevailing perception among youths in West Bengal, prioritizing government jobs over employment opportunities in the private or development sectors, further contributes to the unemployment challenge.

Educational status appears to play a crucial role in influencing the likelihood of experiencing depression. Postgraduate and MPhil/PhD individuals exhibit a higher propensity for mental health symptoms compared to those with a graduation-level education^5,6^. This could be attributed to factors such as heightened academic and career expectations, increased competition in the job market, and potential stress associated with advanced studies^36,37^.

In terms of migration factors, second-time migrants demonstrate a significantly higher level of depression and anxiety compared to first-time migrants^38,39^. Second-time migrants are assumed to bear a double burden of hardships in securing employment, leading to elevated mental health issues^40^. Interestingly, long-term migration did not exhibit a significant association with mental health outcomes. Research indicates that short-term migrants face various compulsions related to family and adjustment to the new environment, which serve as explaining factors for the higher incidence of mental health issues compared to long-term migrants.

The study initially adopted the null hypothesis, suggesting no association between migration, employment status, and mental health issues among higher-educated youths. However, upon analysis, the study ultimately rejected the null hypothesis, revealing a robust association. Migrant youths without employment status are found to be more prone to experiencing mental health issues.

### Limitations of the study

Though this study has some strengths, including the use of the DASS-21 as measure of depression, anxiety and stress, but there are some limitations as well. The cross-sectional design hinders exploring causal links between mental health issues and socio-demographic factors among higher-educated migrant youth. Reliance on self-reported questionnaires introduces potential biases, including method bias, recall bias, and social desirability biases. Future studies could address these through alternative methodologies. The relatively small sample size is a recognized limitation. Additionally, the present study was conducted in Kolkata City, such that generalization may not necessarily be possible.

### Implications of the study

This study is a pioneering effort in Kolkata City, West Bengal, focusing on the prevalent mental health challenges among higher-educated migrant youth. Reflecting global trends, a significant number of young individuals struggle with the transition into their professional lives after completing their studies, placing them at an elevated risk of psychological distress, including depression, anxiety, and stress^41–43^. Recognizing that mental disturbances can detrimentally impact productivity, overall well-being, and escalate the risk of suicide and suicidal behaviors is crucial^44–46^. There is a pressing need for dedicated attention to the mental health of unemployed and underemployed graduates, requiring a thorough evaluation of predictive risk factors through extensive and longitudinal interventional studies.

Encouraging private sector financial investment is imperative. Simultaneously, our nation's youth must be adequately prepared for the future labor market, aligning with the fourth and fifth industrial revolutions. To achieve this, a collaborative effort between the Government, Academia, and Industry is paramount. Technical, polytechnic, and vocational education institutions can play a pivotal role in reducing unemployment by providing relevant training and ensuring graduates secure employment aligned with their skills through improved education and job market alignment. Moreover, in response to the high demand for technically and vocationally trained graduates, job opportunities can be expanded not only within the country but also internationally, drawing inspiration from successful initiatives, such as those seen in China.

## Conclusions

The present study reveals that unemployed graduate youth exhibit higher levels of depression and anxiety compared to their employed counterparts. Additionally, it highlights the high prevalence and associated risk factors of depression and anxiety among job seekers. But the symptoms of stress were more prevalent among the employed because many of the youth were contractual position and due to the heavy workload. These findings underscore the need for preventive workforce initiatives that better align educational channels with job markets. Furthermore, there is a call for early mental support and resilience training programs during higher education to potentially mitigate the elevated risk of mental health issues among graduate youth in Kolkata City, West Bengal. The study suggests the incorporation of skill-based, job-oriented, and professional courses at the graduation to ensure that graduates are better equipped with opportunities for employment. Further exploration with a national large-scale sample is recommended to delve deeper into the identified risk issues.

## Data Availability

The datasets generated during and/or analyzed during the current study are available from the corresponding author on reasonable request.
